# Neuroscience as an Insightful Decision Support Tool for Sustainable Development

**Published:** 2019-10

**Authors:** Rui ZHAO

**Affiliations:** Faculty of Geosciences and Environmental Engineering, Southwest Jiaotong University, Chengdu, 611756, China

## Dear Editor-in-Chief

Decision-making plays in central role in assuring the progress of sustainable development (1). However, it is limited to decision makers' cognitions, given rise to unintended consequences (2). Neuroscience addresses this gap to assist decision making by research on brain function and its ability to change in response to distinct environments. Such a user centered approach may better understand individuals' perceptions and preferences, to lead the decision making process more transparent (3). Neuroscience for sustainable development focuses on the individual's decision-making as a dynamic process that involves economic, environmental and social considerations. Such an approach to understanding decision-making could illuminate people's conscious and subconscious behaviors in response to sustainable development.

Three examples of its practical application are provided to help future research designed to evaluate public's emotional engagement in sustainable development. The first focuses on the question of public acceptance of a transition to renewable energies. An uninformed public's perception of renewable energy systems has become a critical challenge to the adoption of viable applications (4). A decision-making process that does not include consideration of individual risk perception could create significant obstacles to the progress of renewable energy projects. Although traditional psychological research methods—such as questionnaires, surveys, or in-depth interviews—have been widely used to explore the public's willingness or acceptance of certain initiatives, they may prove limited if they fail to capture responses based on perceptions or emotions (5). Neuroscience addresses such gap with an approach that takes irrational fears, prejudice and emotions into account. Moreover, it is methodologically designed to identify the conscious and subconscious responses that correspond to those fears or prejudices, providing a better understanding of the motivations that shape decision-making. An individual's responses may be measured by electroencephalography (EEG) and electrocardiography (ECG) in a visual virtual reality (VR) environment. Brain waves are recorded as the subject experiences the power generation operations of a specific renewable energy project. These neuro signals would serve to distinguish between the subjects' conscious and unconscious responses, thereby providing a greater understanding of their acceptance—or rejection—of the project.

The second example explores efforts to use environmental labeling. An understanding of the motivating factors to purchase products that are environmentally labeled could provide greater insights into sustainable consumption. In general, people believe that in order to mitigate environmental impacts, pro-environment actions are needed. Yet, they may ignore the fact that many consumer practices are irrational with regard to environmental concerns (6). The attention that neurosciences devote to irrational or unconscious decision-making illuminates such discrepancies between social consciousness and actual behaviors. An approach could offer insights into consumers' purchasing practices with regard to environmentally friendly products as well as an understanding of ways that labeling could be changed to achieve a greater impact on the public.

The third example is that of eco-design. Neuroscience could help designers to examine users' satisfaction and/or comfort with products or services that adhere to guidelines for sustainability. Designers usually justify their works based on cost, environmental specifications or functionality (7). However, it proves difficult to quantify the engagement and effect of their products on human psychology and physiology. Neuroscience is capable of identifying the effects of a design on user experiences by decoding their associated brain processes. A possible useful indicator would be the identification of activity in the ventral striatum using functional magnetic resonance imaging (fMRI). Tests could obtain information about the subjects' desires and/or preferences, such as colors, shapes, or aesthetics, to assure the optimal design of a product (8).

Besides, there are numerous ways that neuroscience could be applied to further sustainable development initiatives. Human beings play a critical role in sustainable development through their decision-making and reactions—positive or negative—to initiatives (9). Neuroscience offers a tool by which to evaluate brain processes involved in decision making. Sustainable development efforts could be enhanced by a better understanding of those processes. With the development of sensor technology and wireless communication, neuro-scientific methods coupled with more traditional physiological measures are increasingly capable of monitoring individual behavioral responses. By such shift in focus from external perceptions to internal brain process, greater understanding of peoples' responses could be better adopted to further sustainable development goals

## References

[1] Waas T Hugé J Block T Wright T Benitez-Capistros F Verbruggen A (2014). Sustainability assessment and indicators: Tools in a decision-making strategy for sustainable development. Sustainability, 6: 5512–5534.

[2] Schwarz N (2000). Emotion, cognition, and decision making. Cogn Emot, 14: 433–440.

[3] Venkatraman V Huettel SA (2012). Strategic control in decision-making under uncertainty. Eur J Neurosci, 35: 1075–82. 22487037 10.1111/j.1460-9568.2012.08009.x PMC3325517

[4] Stigka EK Paravantis JA Mihalakakou GK (2014). Social acceptance of renewable energy sources: A review of contingent valuation applications. Renew. Sust Energy Rev, 32: 100–106.

[5] Khushaba RN Wise C Kodagoda S Louviere J Kahn BE Townsend C (2013). Consumer neuroscience: Assessing the brain response to marketing stimuli using electroencephalogram (EEG) and eye tracking. Expert Syst Appl, 40: 3803–3812.

[6] Kollmuss A Agyeman J (2002). Mind the gap: Why do people act environmentally and what are the barriers to pro-environmental behavior?. Env Edu Res, 8: 239–260.

[7] Zhao R Neighbour G Deutz P McGuire M (2012). Materials selection for cleaner production: An environmental evaluation approach. Mater Des, 37: 429–434.

[8] Hubert M Kenning P (2008). A current overview of consumer neuroscience. J Consum Behav, 7: 272–292.

[9] Phelps EA Lempert KM Sokol-Hessner P (2014). Emotion and decision making: multiple modulatory neural circuits. Annu Rev Neurosci, 37: 263–287. 24905597 10.1146/annurev-neuro-071013-014119

